# Decision-making and autonomy among participants in early-phase cancer immunotherapy trials: a qualitative study

**DOI:** 10.1186/s12885-024-12119-7

**Published:** 2024-03-25

**Authors:** Jonathan Avery, Jennifer A.H. Bell, Khotira Baryolay, Gary Rodin, Rinat Nissim, Lynda G. Balneaves

**Affiliations:** 1 School of Nursing, University of British Columbia, Vancouver, BC Canada; 2 Department of Supportive Care Research, Princess Margaret Cancer Centre, University Health Network, Toronto, ON Canada; 3 Department of Clinical and Organizational Ethics, University Health Network, Toronto, ON Canada; 4 The Institute for Education Research, University Health Network, Toronto, ON Canada; 5 Department of Psychiatry and Dalla Lana School of Public Health, University of Toronto, Toronto, ON Canada; 6 Princess Margaret Cancer Centre, Department of Supportive Care, Research Division, 700 Bay St., 23rd Floor, Toronto, ON M5G 1Z6 Canada; 7 Global Institute of Psychosocial, Palliative and End-of-Life Care (GIPPEC), Faculty of Medicine, University of Toronto, Toronto, ON Canada; 8 Department of Psychiatry, Faculty of Medicine, University of Toronto, Toronto, ON Canada; 9 Cancer Experience, University Health Network Cancer Program, University Health Network, Toronto, ON Canada; 10 Princess Margaret Research Institute, Princess Margaret Cancer Centre, University Health Network, Toronto, ON Canada; 11 Rady Faculty of Health Sciences, College of Nursing, University of Manitoba, Winnipeg, MB Canada

**Keywords:** Research ethics, Patient decision making, Cancer clinical trials, Early-phase trials, Qualitative research, Cancer immunotherapy

## Abstract

**Background:**

Participants considering early-phase cancer clinical trials (CTs) need to understand the unique risks and benefits prior to providing informed consent. This qualitative study explored the factors that influence patients’ decisions about participating in early-phase cancer immunotherapy CTs through the ethical lens of relational autonomy.

**Methods:**

Using an interpretive descriptive design, interviews were conducted with 21 adult patients with advanced cancer who had enrolled in an early-phase CT. Data was analyzed using relational autonomy ethical theory and constant comparative analysis.

**Results:**

The extent to which participants perceived themselves as having a choice to participate in early-phase cancer immunotherapy CTs was a central construct. Perceptions of choice varied according to whether participants characterized their experience as an act of desperation or as an opportunity to receive a novel treatment. Intersecting psychosocial and structural factors influenced participants’ decision making about participating in early-phase cancer immunotherapy trials. These relational factors included: (1) being provided with hope; (2) having trust; (3) having the ability to withdraw; and (4) timing constraints.

**Conclusions:**

Findings highlight the continuum of perceived choice that exists among patients with cancer when considering participation in early-phase cancer immunotherapy CTs. All participants were interpreted as exhibiting some degree of relational autonomy within the psychosocial and structural context of early-phase CT decision making. This study offers insights into the intersection of cancer care delivery, personal beliefs and values, and established CT processes and structures that can inform future practices and policies associated with early-phase cancer immunotherapy CTs to better support patients in making informed decisions.

**Supplementary Information:**

The online version contains supplementary material available at 10.1186/s12885-024-12119-7.

## Background

Advances in cancer care depend on evidence derived from clinical trials (CTs) to help practitioners and patients make informed treatment decisions. However, less than 5% of adult patients with cancer participate in CTs [1] and many trials suffer from insufficient accrual, with earlier phase trial being especially problematic [2]. Poor accrual and retention have been linked to complex and lengthy consent procedures, which may create difficulties for patients in providing informed consent [3, 4]. A systematic review evaluating patient understanding of consent for participation in CTs concluded that patients often do not fully understand the potential health risks of novel experimental drugs and have unrealistic expectations of benefit [5].

Phase I trials, which test the toxicity and safety of a novel treatment in humans, have typically placed research subjects at greater risk of adverse effects with less likelihood of benefit compared to phase II and III trials [6]. Phase I cancer trials usually involve patients whose disease is advanced or refractory to standard treatment, and who may be more likely to view the CT as their last opportunity to receive treatment [7]. Such individuals may feel desperate about the severity and progression of their disease, which can undermine their capacity to provide meaningful informed consent [8, 9].

Recent advances in our understanding of cancer biology and the emergence of precision medicine, with such transformative therapies as immune checkpoint inhibitors and molecularly targeted agents, have altered the cancer CT landscape [10]. Some phase I cancer trials have become highly targeted and are incorporating elements of later phase CTs. It is not uncommon now to have a combined phase I/II within a single study protocol. These early phase CTs include dose expansion, and allow modification of the trial design based on early evidence of a drug’s efficacy and acceptable toxicity [11]. These combined phase I/II trials [12] raise ethical concerns as the distinctions between trial phases becomes blurred, challenging previous understandings of the risks and benefits associated with phase I trials while at the same time offering participants a renewed sense of hope for a cure or delayed disease progression [13]. Early phase trials thus require special attention to informed consent procedures to ensure research subjects’ understanding of evolving safety and efficacy data, and realistic assessment of risks and benefits [11].

Given the ethical concerns associated with these modern early-phase CTs, the purpose of this qualitative study was to explore the decision-making process of patients living with cancer who have considered participating in early-phase cancer immunotherapy CTs, including the psychosocial (i.e. the interrelation of social factors, such as age and socio-economic status, with individual thought and behavior) and structural factors (i.e. inequities and unjust power differences in policies and practices) that intersect and influence perceptions of choice and ability to provide informed consent. Consideration of the dynamic influence of psychosocial and structural factors on individual-level decision making is essential to the development of a richer and more comprehensive understanding of CT decision-making processes. This knowledge can enhance ethical CT practices and the development of associated tools, resources, and policies to support patients in making informed decisions about early-phase cancer CTs in this rapidly advancing and complex trial landscape.

## Methods

### Research design and theoretical framework

A qualitative interpretive descriptive design [14], using semi-structured interviews and constant comparative analysis with a relational autonomy lens [15], was used to explore the intersection of the dynamic social and relational factors influencing patient decision making about early-phase cancer immunotherapy CTs. Relational autonomy was selected as a framework for this study because it is a theoretical approach that, in addition to psychosocial factors, considers the influence of structural factors on an individual’s beliefs and opportunities to develop autonomy, which is necessary for providing informed consent. By identifying the structural factors, such as gender or socioeconomic status, and addressing related inequities and unjust power differences in policies and practices, relational autonomy aims to empower those who may be oppressed [16]. According to relational autonomy theory, a person may be regarded as minimally, medially or fully relationally autonomous based on the degree to which their motivation arises from their own autonomous capacities (i.e., experience a desire as “one’s own”), within an overlapping network of social and structural contexts [17]. Although prior studies have identified psychosocial influences on trial decisions, less attention has been paid to identifying structural factors and the intersection between psychosocial and structural factors on cancer CT decision making [13, 18]. Relational autonomy theory appreciates the intersectionality of these factors and is thus well-suited to guide an examination of the complex interplay of these factors in creating a holistic context in which persons make early-phase cancer CT decisions.

Bell’s method for applying relational autonomy to qualitative health research [15] guided all aspects of this study, including the development of the research question, study design, sampling, and interview guide. Relational autonomy was also used as an analytic lens to uncover and interpret the personal and structural factors that influence individuals’ autonomy and informed consent within the context of early-phase cancer immunotherapy CT decision-making.

### Participants

Participants were recruited from lung, breast, gynecologic, gastrointestinal, brain and CT clinics at the Princess Margaret Cancer Centre in Toronto, Canada between September 2018 and June 2019. The hospital has a world-renowned early-phase CT program, conducting approximately over 400 phase I/phase I-II trials each year, predominantly testing novel anti-cancer agents such as immunotherapies. Convenience sampling was initially used to recruit participants followed by a purposeful sampling strategy to ensure that diverse gender perspectives were represented in the data, as well as individuals who had declined to participate in an early-phase CT. Patients were identified and informed about the study by CT nurses. Interested patients were then approached by a member of the research team to provide further information verbally and in written form about the study and to seek informed consent. The primary inclusion criteria for patients were English speaking individuals aged 18 years and older who had been approached to take part in an early-phase (I or I/II) CT. This study received research ethics approval from the University Health Network Research Ethics Board (#18-5408). All participants provided written informed consent to participate. A token of appreciation in the form of a $25.00 gift card was provided to those who participated.

### Data collection

Semi-structured interviews were conducted with participants during September 2018-June 2019 either in person or over the phone by the first authors (JA, JAHB). We used the theory of relational autonomy to develop the interview guide. Questions were crafted that allowed us to probe the psychosocial (i.e. personal and relational) and larger structural (i.e. macro-level) context that may influence an individual’s autonomy and decision making in consenting to partake in an early-phase CT, and the potential influence of personal and relational factors (Table S1). Additional questions probed participants’ understanding of phase I trials and the purpose of the trial they were offered, reasons why the patient accepted a trial, who and what was influential in their decision-making process, and what information or other resources were important when making the decision. Applying the theory of relational autonomy to develop the interview guide allowed us to explore the context in which the trial was introduced and any perceived influences on making a decision about partaking in the trial (i.e. barriers and facilitators). All interviews were digitally recorded and transcribed verbatim. Before each interview, participants completed a self-reported sociodemographic questionnaire. This data was used to determine the heterogeneity of the sample across age and gender, which directed purposeful sampling strategies to ensure representation of our sample across these two criteria. This was facilitated by the research team asking our clinical partners if they had potential participants between certain ages or genders to fill the gaps in our sample.

### Data analysis

All interviews were transcribed verbatim. Transcripts were analyzed using the constant comparative method to compare all “pieces” of data (codes, themes, and categories) within and across interviews until substantive patterns were developed [19]. In this approach, data collection and analysis are inextricably linked, with the interview data directing the coding and vice versa. A relational autonomy lens was applied to the analysis to examine the personal, social, and structural factors that influenced patients’ decisions about participating in early-phase CTs. The impact of family, the healthcare team, and the larger healthcare system was also explored, as well as how power manifested within these social dynamics. The use of a theoretical framework, such as a relational autonomy, in qualitative research is considered acceptable and does not undermine inductive analysis [20]. Each interview transcript was reviewed line-by-line, and/or in segments, to identify codes. Codes were grouped together into larger themes and corresponding categories. The first four interview transcripts were analyzed independently by three members of the research team (JA, JAHB, KB) and then discussed to create and agree upon a coding framework. Thereafter, the remaining interviews were analyzed by JA.

Consistent with interpretive description and reflexivity is acknowledgement that qualitative research is bound to historical context and the disciplinary perspectives of those involved in the study [19]. Reflexive journaling and memo writing was undertaken by JA to maintain rigour and transparency of the data analysis and emergent findings were discussed with the research team. Each member of the research team was instructed to discuss their thoughts and insights associated with the data analysis and emergent findings to illuminate potential beliefs and biases in the analysis. Any discrepancies between views were discussed and resolved through discussion. NVivo qualitative software program was used to facilitate coding and analysis. The use of rich, descriptive quotes and line-by-line coding further contributed to ensuring the representativeness and trustworthiness of the analysis and findings, as did engagement with the research team members who are active with cancer CTs in Canada and who believed the findings resonated with their experience.

## Results

### Demographics

We approached 50 patients to participate in this study of which 23 consented to participate. Of those who did not participate, six patients did not meet eligibility requirements, eight patients declined the offer of study participation, and 13 did not respond to follow-up by the research team. Data was not collected on why participants declined to participate. Of the 23 who consented to participate, two individuals withdrew from the study leaving a final sample of 21 patients who were interviewed by telephone (*n* = 11) or in-person (*n* = 10) in a private space at the cancer centre. On average interviews were 50 min in duration (range: 22– 70 min). We did not note any differences in depth or length between interviews conducted in person versus those conducted by telephone. All the patients interviewed, except one, had accepted a phase I or phase I/II cancer CT of immunotherapy. One individual took part in a radiotherapy phase II trial. The predominance of immunotherapy trials reflects the current priorities of the CT program at the host institution. Despite repeated attempts, we were unable to recruit a larger number of participants who had declined an invitation to participate in an early-phase CT; however, some had previous experience withdrawing from a trial or declining a trial in the past. Just over half of the participants identified as male. Most participants identified as White and reported having at least a college/university education and a yearly income of $30–60,000 Canadian. The patient sample varied by cancer type (Table S2).

### Findings

All participants considered participating in an early-phase CT. However, the extent to which they perceived themselves as having a *choice* to participate or not emerged as a central construct of their experience. Participants’ perceptions of choice varied according to whether they characterized their experience as either: (1) an act of **desperation** when standard care was seen as unsuccessful and/or no longer an option; or (2) an **opportunity** to receive a novel treatment to potentially improve their quality of life, increase their longevity, and/or be cured. These divergent perceptions exemplify opposite ends of a choice spectrum, where desperation coincided with the sense of having less choice, and opportunity afforded participants a sense of greater choice (see Table S3).

### Trial participation as an act of desperation

Despite research consent forms that identify alternative options to trial participation, some participants believed they did not have a choice but to participate in an early-phase cancer CT. These participants reported that standard care (i.e., chemotherapy, radiation and/or surgery) was no longer effective in treating their cancer, or the side effects of these treatments were intolerable or too difficult to manage, thereby negatively impacting quality life. A minority reported never being presented with other treatment options by their oncology healthcare team because of the severity of their disease. When faced with a life-limiting diagnosis and no viable treatment, participants perceived they had no other option but to enroll in an early-phase trial. Such individuals did not believe that declining the trial was an acceptable option. As one woman with colon cancer said:

My understanding is they [CT staff] don’t really know if it’s going to help me. They definitely told me that it wasn’t a cure, but the chemo really wasn’t working… and when you’re told this is terminal, what are you going to do? There’s no other road as far as I myself am concerned. We’re out of options. (P007)

The belief that death was around the corner caused a great amount of anxiety in some participants, contributing to their sense of desperation for trial participation, even when faced with potential side effects from the drug being trialled. One woman with cervical cancer emphasized:

… it’s very scary. I was petrified of what the side effects could be [from the trial] but again you don’t have a choice. If you want to stay here [stay alive], you’re going to do it (P010).

### Trial participation as an opportunity

In contrast to individuals who felt a sense of desperation about gaining access to a trial, there were those who perceived themselves as having a choice. While they held hope that an early-phase trial could help them, they expressed more equanimity about the outcome and felt able to critically evaluate their treatment options and the impact on their overall wellbeing. For many of these individuals, early-phase cancer immunotherapy CTs were understood as an opportunity to try a novel therapy that could provide a better quality of life when compared to standard forms of treatment. A woman with breast cancer said:

I mean chemo, when I looked at the chemo side effects and also the clinical trial side effects, they’re all so similar. I figured, you know what, why not just give it [the trial] a chance because we’ll never know until I give it a try… I just feel like this is something that could work (P005).

Other participants focused on the promise offered by an early-phase trial and contrasted it with the known risks, side effects, and limited success rate of standard treatment for their disease. For example, a woman with lung cancer shared:

You just hear horror stories about people on chemo. Do I want to put myself through hell… to be taken to the point of death for something that’s not a cure? (P013)

Logistics associated with participating in an early-phase CT, such as travelling to the cancer centre for treatments and follow-up care, were also considered by some when making a trial decision. Most participants who felt they had a choice appeared to weigh the pros and cons of participating in a trial regarding the impact on their daily routines, lifestyle, and on their desired quality of life. This type of decisional balancing is reflected in the following quotation from a man with lung cancer:

I felt I had a choice. I was offered the trial, but I didn’t accept right away. I was reluctant. There was a greater commitment [to participate] and perhaps a greater risk of side effects. My big concern was quality of life. As long as I can still think and write… I’m good… this seemed like an opportunity. (P016)

Applying a relational autonomy lens, this quote suggests that those who saw themselves as having a choice were better positioned to reflect and consider the impact of a trial on their lives and health. Those who perceived themselves as having less choice, may not have had the luxury to consider comprehensively the impact on themselves.

### Relational influences on early-phase trial decision making

A myriad of psychosocial and structural factors, reflecting the relational nature of early-phase CT decision-making, influenced participants’ decisions about participating in early-phase cancer immunotherapy trials. These factors included: (1) being provided with some hope; (2) having trust; (3) having the ability to withdraw; and (4) timing constraints. In constructing these factors, it became apparent that all participants, even those who perceived a lack of choice, were able to exercise some degree of relational autonomy within the psychosocial and structural context of early-phase trial decision making.

#### Being provided with some hope

Foremost, participants’ decisions about participating in an early-phase cancer immunotherapy trial appeared to be influenced by their belief that the trial intervention would be effective in potentially extending their life and preserving their quality of life, which contributed to an overarching sense of hope. Important social relationships and structural influences, including the larger cancer care landscape, were influential in the maintenance of hope, which was an important relational factor that influenced participants throughout the choice continuum.

With regards to the impact of one’s social relationships, many patients experienced encouragement from their family and friends to persevere in the face of a dire prognosis– early-phase trial participation was seen as a logical next step in continuing “the fight” and maintaining hope. A woman with cervical cancer said:

You know what, my husband has been very, very strong and I know he’s…this is very, very hard for him because I try to put myself in his shoes to be the one that gets left behind. He’s been strong and I know it’s killing him inside. Now, my boys, they think mum’s strong, she’s coming out of this, like, they just…they won’t accept it either…they’re just of the belief that mum’s going to make it through this. I mean, decisions were made because of them, I can tell you that. (P010)

Considering this woman’s experience through a relational autonomy lens leads to further questions that could be explored related to how the social norms of gender identity influence CT decisions. Specifically, whether the woman was encouraged by her family to pursue the trial or whether her decision was circumscribed by a sense of responsibility to not leave her family members behind, stemming from social expectations regarding gender roles and motherhood.

In addition, although conversations between clinicians and patients were transparent about the experimental nature of early-phase cancer immunotherapy CTs, they often provided participants with hope that the trial would be beneficial. The body language, tone of voice, and choices of words when trials were introduced all influenced patients’ optimism about the outcomes of a trial. For example, some described receiving information about the uncertainty of personal benefit from the trial, yet remained hopeful that the trial could potentially extend their quality of life. As stated by a woman with colon cancer,

The doctors themselves, they don’t know if it’s going to help. They definitely told me it [the trial] wasn’t a cure, but it could give me more time. Hopefully. It was an alternative to chemo. It was kind of like, hey, I can handle that because I’m not feeling the nausea and I have the energy to actually live and go and do things. (P007)

The introduction or maintenance of hope through interpersonal relationships gained momentum from larger structural influences, such as the practice of introducing early-phase CTs to individuals shortly after receiving a dire prognosis. Given the devastating psychological impact for participants of being told they were out of options and could possibly die, the introduction of highly experimental treatments provided them with a renewed sense of hope that they still had a chance to live. A man with lung cancer said:

When you’re given a cancer diagnosis, I mean, I was told I had four months [left to live], that’s kind of a shock. And then you’re told that maybe you have a new lease on life by [Nurse X] about going on this trial! (P017)

Participants clung to this sense of hope in moments of despair and were highly influenced by it when considering participating in an early-phase CT.

Participants also appeared to derive a sense of hope from the numerous medical tests that were required to determine if they were a candidate for a trial. These tests made the trial appear highly personalized and, depending on the results, eliminated any hesitancy about taking part. A man with leukemia said:

I did a bunch of tests, so they knew how good or bad of a candidate I was and they said, ‘you’re like, a perfect candidate’. So, it was a no brainer [to participate] (P015).

This example illustrates how the structural context of trial work-up, reinforced by the positive framing of test results by CT personnel, contributed to patients’ trial decisions.

The experience of hope also appeared to be heightened by social expectations regarding the new trial landscape of breakthrough therapies, including immunotherapy agents. A man with sarcoma discussed how these therapies were less toxic than other cancer treatments and leveraged the body’s natural immune system:

It’s using your own immune system so it’s more of a natural approach than chemo. Chemo is a chemical, it’s poison […] and the idea is that they give you the chemical and it kills all the cells including the good ones and the idea is the hope that you bounce back. (P002)

#### Having trust

Trust played an important role in encouraging participants to consent to an early-phase CT. Foremost, the faith and trust patients had for the clinical institution in which the trial was being offered was an influential factor in the decision to take part and increased their confidence in the safety of the trial. A woman with ovarian cancer said:

Initially, I was very hesitant to participate but I have great faith in [Hospital X]… they’re not going to introduce a phase I [trial] if there’s going to be a high degree of danger. (P020)

Participants also felt assured that the institution would look out for their best interests. As a man with colon cancer noted:

I’m not a 100% sure about them [early-phase clinical trials]. But I know that [Hospital X] is highly reputable… they’re going to take care of me. They are going to keep a close eye on how things are going and if things go bad, they will stop the treatment. I was just very excited that I was able to get to [Hospital X]. (P003)

In addition to trusting the institution, participants were also influenced by the opinions of trusted CT staff and other members of their healthcare team. A man with leukemia said:

[Doctor X] offered me [the trial] and when I think about it, I trust [Doctor X] and his integrity and I trust his beliefs that this [the trial] is the best choice for me… I followed. (P009)

Another man with pancreatic cancer reflected on how the history of successful treatment recommendations provided by his oncologist influenced his trial decision:

I wanted to talk to him so I could get his input because he’s important and he’s kept me going this long. He said, ‘Go ahead with it, you’re foolish not to take it’. (P011)

The use of the word “foolish”, while potentially reflecting the established and comfortable rapport between the patient and the clinician, could have also made it challenging for the patient to do anything but accept their physician’s recommendation to participate in the trial.

#### Having the ability to withdraw

The regulatory and ethical requirement that allows patients to voluntarily withdraw from a CT at any time was a structural factor that appeared to facilitate participants’ decisions related to early-phase trials. Both participants who felt desperate and those who perceived participating as an opportunity described the ability to withdraw as a ‘failsafe’, making it easier for them to ‘take the risk’ of participating in an early-phase trial where there is more uncertainty about safety and efficacy. A man with leukemia elaborated:

I wanted to know whether I could quit at any time. I didn’t want to just be a guinea pig unless I had a chance of survival and some kind of potential improvement. (P014)

Similarly, a man with lung cancer who felt he had no choice but to participate, appeared comfortable with his decision because he had the option of withdrawing from the trial, saying:

The bottom line is I didn’t think I had any choice… but it’s nice to know that I could have walked away at any time. (P018)

#### Timing constraints

There were both real and perceived timing constraints expressed by participants in relation to decisions about taking part in early-phase cancer immunotherapy trials. Structurally, CTs can have a limited time frame to recruit subjects and eligibility criteria can restrict access to individuals within a narrow window of time in the cancer continuum and with regards to pre-screening. As such, some participants internalized a sense of pressure to make a quick decision, lest their spot in the trial would be filled by someone else. This contributed to their feelings of desperation and perceived lack of time to make a considered choice. A man with pancreatic cancer said:

There was pressure to make the decision in a hurry… they [CT staff] have to fill these spots… they only have a certain amount of time for you to make the decision. Eventually, you’re going to get to the point where we have no choice. You have to go for a clinical trial. (P011)

Some participants also perceived themselves to personally be under a time constraint to participate in an early-phase cancer immunotherapy trial with regards to gaining access to a novel treatment while they believed benefit was still possible. However, the nature of early-phase CTs, which are typically reserved for individuals with refractory disease who have been provided all treatment options, creates a structural barrier to those seeking early access. For example, a man with sarcoma was informed by their treatment team that early-phase CTs were not yet an option because the patient had not exhausted more proven lines of cancer therapy. Waiting for a trial to become available became a point of contention and distress for this participant, who was desperate to receive the immunotherapy trial drug as soon as possible because they perceived it as the better treatment option that they would benefit from, especially if received earlier in their disease trajectory:

After it [immunotherapy clinical trials] was mentioned, I kept pushing for it. I wanted to try it out. I was willing but the doctors kept saying ‘that’s something to keep your back pocket. That’s for later’. I’m going, ‘well, later on may be too late’. (P002)

## Discussion

This study explored patients’ decision making related to early-phase cancer CTs using the lens of relational autonomy. A choice spectrum was uncovered that captures patients’ decision-making experiences, ranging from trial participation being framed as an opportunity to it being perceived as an act of desperation. Our use of the theory of relational autonomy in the construction of our interview guide allowed us to explore and uncover the personal, relational, and larger structural factors that framed this experience. For example, asking questions pertaining the participants motivations and reasons for accepting/declining/withdrawing their participation illustrated how their clinical team influenced participants’ perceived hope and trust that the trial would extend their quality of life and even provide a potential cure, while structural factors, such as trial design and ethical requirements that ensured voluntariness of decisions, enabled the ability of patient participants the option of withdrawing from a trial should they change their mind. A perceived sense of hope and trust that the trial would succeed, and the ability to withdrawal were fundamental attributes that influenced the decision to partake.

Our study findings align with and expand upon previous research. For example, Halpern et al. [21] illustrate that upwards of 94% of patient-subjects enroll in early-phase CTs with unrealistic therapeutic benefits formed when facing a life-limiting illness that is very difficult to treat. Cox et al. [22] note that beliefs about therapeutic benefit may be influenced by a myriad of factors including how healthcare professionals working in early-phase CT recruitment communicate with potential participants and the discourse used that could impact patients’ perceptions of choice. For example, the use of the term ‘foolish’ to describe a decision not to enter a CT has significant implications for patients’ decision making, as other studies have also found patients are highly influenced by their physician’s communication [23]. Additionally, recent studies have also uncovered that some patients perceived they have no other choice but to participate in the trial, perceiving ‘no treatment’ as an untenable option as it means giving up on life [23, 24]. For those who were ineligible for trials, this led to feelings of despair and uncertainty about their options [23]. More research is needed that explores patients who are ineligible or who choose not to enroll in clinical trials to offer tailored psychosocial support, especially since one study has found a correlation between moderate to high levels of clinical depression and the decision to decline CT participation [25].

Applying a relational autonomy lens allowed us to explore potentially influencing factors in more detail by highlighting the overlapping social and structural factors that affected patients’ ability to enact preferred choices and how they were supported in their preference-formation and decision-making process. Many participants shared that they were approached by their physician about taking part in an early-phase trial immediately following the devastating news of a cancer recurrence, progression of disease, or lack of response to standard treatment. This timing and the framing of early-phase cancer immunotherapy trials as the next logical step in treatment may have limited participants’ ability to make a considered decision about trial participation. Patients’ desperation related to the severity of their disease has been identified in previous literature as autonomy-undermining by interfering with persons’ abilities to clearly consider and reflect upon one’s values, interests, and options [26]. The paradox of obtaining informed consent for early-phase trials in circumstances described by many patients as life versus death, in which they perceive no other options but to choose the trial, has been previously discussed [27, 28]. While the vulnerability of patients must be acknowledged and addressed in this scenario, the nuanced lens of relational autonomy recognizes that individuals may still have capacity for autonomy, although grounded in important social relationships and influenced by structural factors. Therefore, instead of questioning whether patients have the ability to make their own decisions, a relational autonomy lens encourages the healthcare team and CT personnel to consider ways to support patients so that they are more fully relationally autonomous in the early-phase CT decision making-process. For example, patients’ capacity for autonomy within a relational context would be enhanced by CT personnel taking the time to ensure an individual’s emotional and psychosocial needs are met, that their personal values and beliefs are reflected upon during the decision-making process, and that their understanding and expectations regarding trial outcomes are realistic [27, 28].

The larger social discourse surrounding cancer and emerging cancer treatments also had an influence on participants and their decision making related to early-phase cancer immunotherapy trials. When faced with a poor prognosis or limited treatment options and the associated fear and desperation, some patients and family members leveraged bellicose metaphors, such as continuing “the fight” against cancer, to provide hope and to support their decision to enter an early-phase trial [29]. Further, the recent emergence of cancer immunotherapy and precision medicine, and the accompanying discourse regarding the personalized and “natural” immune boosting nature of treatment, has further contributed to patients’ and clinicians’ sense of optimism towards early-phase trials focused on these types of therapies. Acknowledging the powerful influence of these discourses on patients’ decision making and their ability to reflect on their personal values as well as the potential benefits and risks of trial participation may be an important consideration to enhance patients’ relational autonomy in the context of early-phase cancer immunotherapy CTs. There is considerable debate in the scientific and ethics literature about whether phase I trials are likely to provide direct therapeutic benefit in some instances [6, 30, 31]. We do not weigh in on this debate. Instead, we underscore the need for patients to understand the potential benefits and risks of a particular early-phase trial without hyperbole or unreasonable optimism [32]. Specifically, immunotherapy is not without potentially life-threatening risk of adverse events, such as neurotoxicities and uncertainty exists regarding the long-term benefits and remission status of patients receiving these therapies [33]. It behooves physicians when discussing immunotherapy or other targeted cancer CTs to remain committed to providing balanced communication with patients about the purpose of a trial and to clearly explain the associated risks / benefits so as not to engender unrealistic expectation and provide false hope [23]. Additionally, those who contribute social commentary and public-facing information about novel cancer drugs and CTs such as the media, industry, and trusted cancer centres and organizations, need to be held accountable (perhaps by regulation if not by ethical standards) to present information about modern day CTs fairly, without the use of unjustified superlatives or emotional advertising that obscures a more full appreciation of the risks, benefits, and direct participant costs/resources [34, 35].

Examining the study findings through a relational autonomy lens highlights the power differential between patients and clinicians, and its potential impact on patients’ perceptions of choice. The trust and sense of indebtedness that many patients feel towards their oncologists, as well as the institution in which they are receiving care, may restrict patients from fully considering their own personal values and preferences when making an early-phase trial decision. In addition, the immutable expertise and scientific knowledge held by oncologists may be prioritized by some patients and motivate them to accept a clinical recommendation to participate in an early-phase trial. The structural location of oncologists as the “gatekeeper” to early-phase CTs may also contribute to a power imbalance that restricts the choices available to patients and families [36]. In addition, it is important to acknowledge that clinicians and institutions are under pressure and may feel a sense of responsibility to successfully accrue to early-phase trials, not only to contribute to the scientific evidence but to provide patients with the latest advances in cancer treatment [36].

Applying a relational autonomy lens in this study also uncovered how structural factors, including the design and pre-screening processes associated with an early-phase trial, impacted patients’ decisions and their perceptions of choice. It is important that CT personnel and healthcare professionals recognize how the restrictive eligibility criteria, extensive screening procedures, and conversations about trial participation unintentionally characterize early-phase cancer immunotherapy trials as exclusive and time-limited, which may lead some patients to feel pressure to accept the trial as well as form unrealistic expectations and hope for personal benefit. Successfully making it through the comprehensive early-phase trial screening may also create a situation in which patients feel the trial is tailored to them and that refusing to take part would be nonsensical. Healthcare professionals and CT personnel, thus, need to be aware of how the very structure of immunotherapy or targeted early-phase trials may inadvertently influence patients’ decisions related to trial participation. Being clear in conversations with patients that being an eligible candidate for an early-phase trial does not necessarily equate with receiving direct benefit, including an extension of life, may be an important addition to discussions about trial participation.

In accordance with interpretive description study methodology, we sought to not only interpret what was stated by our study participants, but also to recognize the silences in the data, or what was not alluded to and constituted a marked absence from what may have been expected in the data. Notably, when participants were asked about what was discussed when considering early-phase immunotherapy CT participation, as well as what type of information was important to them, the topic of advance care planning did not emerge at all. Further exploration of how advance care planning and goals of care conversations are managed in this context, and how patients can be supported in considering and evaluating all possible treatment and care options, including palliative care, may be a valuable line of future inquiry. This finding aligns with research that has identified a divergence between patients receiving specialist palliative care and those considering an early-phase trial that is based in misconceptions about palliative care as only applicable near the end of life [37]. The heavy physical symptoms and psychological burdens that many patients face is highly relevant to the services of specialist palliative care, and it has been argued that earlier education be provided to patients and caregivers to explain the role and opportunity for palliative care throughout the cancer trajectory [38]. However, when prognosis changes and end of life is near, the social and structural context of cancer care needs to more fully embrace end-of-life care as an acceptable and appropriate option for some individuals, countering the perception of palliative care at this stage of disease as a clinical “failure” [39]. CT personnel thus need to work closely with a patient’s healthcare team to ensure meaningful discussion about palliative care occur alongside or prior to early-phase trial recruitment procedures. Such discussions will support patients’ re-evaluation of their priorities related to quantity of life versus quality of life, create space for patients and their family members to consider the full spectrum of healthcare alternatives, and enable patients to make a more fully informed and relationally autonomous decision.

## Limitations

This was an interpretive descriptive qualitative study undertaken with a cross-sectional design. It is important to acknowledge that the study design restricts our ability to make inferences regarding how the personal, social, and structural factors influence where a participant was located on the choice spectrum with regards to perceiving their decision to participate in an early-phase trial as being an act of desperation or one of opportunity. Future research utilizing other methodologies, such as grounded theory or survey research, may provide further insight into the relationships between these factors and choice perceptions.

In addition, despite our best efforts, we were not able to recruit participants who had withdrawn from, or declined the offer to join, an early-phase CT. Several participants, however, did have experience in declining a CT offer in the past. As such, the study findings must be cautiously applied to all individuals engaging in decisions about early-phase trial participation as the study sample may have captured a select population with regards to attitudes and beliefs towards clinical research and end-of-life care, as well as their overall decision-making experience. Further, the study was conducted at an urban, academic cancer centre with a highly resourced and active early-phase CT program - the experience of individuals living in rural or remote regions and receiving care from community-based oncology programs may be quite different. The study sample, however, was diverse with regards to gender identity, type of cancer, and yearly income.

Lastly, Canada’s commitment to universal health care and the associated stewardship constraints with regards to approving and funding new cancer therapies creates a unique context regarding the role of early-phase CTs in providing individuals access to novel and expensive treatments. Replicating this study in a non-single payer healthcare system, such as the United States, may offer additional insights.

## Practice and policy implications

In the context of presenting an early-phase trial to patients, all clinicians and CT personnel need to be meticulous in their language and how they frame and present such trials, including how they discuss screening procedures and the suitability of a person given the trial’s eligibility criteria. Specifically, there must be an explicit description that the trial is research, not treatment, that the benefits are unknown, and the risks are still being understood. In addition, care is needed so that the often-extensive screening procedures associated with early-phase trials (i.e., blood work, biomarker testing, MRI/CT scans) do not create what has been called “hype and hope” [40], in which patients do not fully appreciate the potential for harm, including having their quality of life suffer for unknown benefit.

From both a practice and policy perspective, it is imperative that the power imbalances that exist between patients and physicians be acknowledged in early-phase CT accrual. There is enormous trust and confidence placed on physicians and their advice; the perception that one’s physician is enthusiastic about a trial and supportive of participation may unintentionally influence patients’ decisions. As such, it is not enough to simply remind patients that their participation in an early-phase trial is voluntary and they can withdraw at any time; instead, a more in-depth conversation needs to occur about their goals of care, where they are in the cancer trajectory, and the social and structural influences that may impact patients’ decisions. For example, unpacking the complex relationship between clinical care and research and how members of the healthcare team may have dual roles as clinicians and researchers may be important to clarify with patients to support them in making a fully informed decision. Guidelines and training for physicians and CT personnel are also required to promote reflection of how power and influence is manifested in early-phase trial discussions and provide them with the skills and insight to have nuanced conversations that support patients’ relational autonomy in the decision-making process.

One way that the power imbalance between patients, physicians, and CT personnel could be addressed is by co-creating opportunities with patients and patient advocacy groups for greater engagement in the planning and design of early-phase CTs. By including patients as equal partners throughout the trial continuum, from conception to translation, not only will power be better distributed, but problematic issues related to patients’ relational autonomy can be identified and addressed. Such an approach has been put forth by both patient advocacy groups (e.g., Colorectal Cancer Canada) as well as national (e.g., Canadian Cancer Trials Group, N2 Canada) and international research alliances (Network Institute of Health and Care in the United Kingdom). These initiatives promote transparency and education regarding the intent, nature, and potential benefits and risks of CTs among patient communities, as well as increase awareness of on-going trials. Such engagement may also lead to more patient-oriented and pragmatic trial designs that allow a more diverse population of individuals to be recruited and retained in trials, and the inclusion of person-centred outcomes that may reflect the needs and values of patients. A national summit to discuss the ethical challenges posed by modern day early-phase trials is urgently needed, especially for trials of breakthrough therapies (i.e., immunotherapy), whose early promising results can create pressure to rapidly implement costly therapies with limited long-term efficacy and safety data into clinical practice.

## Conclusion

Early-phase CTs are an essential step in the development and implementation of new cancer therapies that provide hope to patients and their families who have reached an impasse in their curative treatment journey. In this challenging time, coupled with the potential “renaissance” of new and emerging targeted experimental therapies [41], it is imperative that patients are supported in their ability to make a fully informed decision about trial participation, without undue influence from social and structural factors. A relational autonomy lens encourages us to recognize the complexity of the early-phase trial decision-making process and view the patient not as a vulnerable individual incapacitated by despair and grasping for straws, but as someone who is to be supported and empowered by their healthcare team and CT personnel.

### Electronic supplementary material

Below is the link to the electronic supplementary material.

Supplementary Material 1: Interview Guide

Supplementary Material 2: Participant Characteristics

Supplementary Material 3: Themes and Supportive Quotes

## Data Availability

The datasets analysed during the current study are available from the corresponding author on reasonable request.
