# Supplementary material for: Decision-making and autonomy among participants in early-phase cancer immunotherapy trials: a qualitative study
Source: BMC Cancer. 2024 Mar 25;24:373. doi: 10.1186/s12885-024-12119-7 (PMC10962144; doi:10.1186/s12885-024-12119-7)
Supplement: Supplementary file 1 — Supplementary Material 1: Interview Guide [file 12885_2024_12119_MOESM1_ESM.docx]

Supplemental File

**Interview Guide: Cancer Patient Autonomy & Decision-Making in Early-Phase Trial Recruitment**

| **Area** | **Questions** | **Probes** |
| --- | --- | --- |
| 1. Context | - Can you tell me briefly a bit about yourself - when you were first diagnosed? What has been your experience with cancer to date? - Who approached you about taking part in a Phase 1 clinical trial? - What is your understanding of the purpose of Phase 1 clinical trials? - What is your understanding of this particular phase 1 trial that you were offered? | - What treatments or clinical trials have you done in the past? - How was the trial presented to you? - Who else was present during the conversation about the trial? - Probe therapeutic benefit, first-in-human trial, and understanding of targeted therapy/immunotherapy - what drug is it testing, what part would you have been involved in – dose escalation/expansion |
| 1. Influences on Decision Making | - Because the trial is Phase 1 there aren't many people who have taken this drug before. How/if did this influence your decision-making process? - What were the reasons you decided to accept/decline/withdrawal the phase 1 clinical trial? - In thinking about the most recent Phase 1 trial you were offered: What information was important to you in making this decision? - On a phase 1 trial some of the risks of side effects are very severe. How did you weigh risks versus benefits within your decision-making process (e.g. risk of mortality, major organ damage, neurological damage)? - How informed do you feel you were in making this decision? - Thinking back to the study materials and the consent form you read, how informed did you feel after reviewing the materials? - On a scale of 1 to 10, where 10 represents “totally informed” and 1 represents “barely informed”, how would you rate your experience of consent? Can you elaborate? - Who did you involve in your decision making? Family members? Health professionals? Others? - How involved were you in the decision to take part in the trial? - How was your decision about being part of a Phase 1 clinical trial informed by your previous health care experiences? Previous treatment decision-making experiences? | - To what extent did you consider information about the impact of trial participation on your cancer? - To what extent did you consider information about impact of trial participation on your quality of life? - To what extent did you consider information about impact of trial participation on your relationships? - To what extent did you consider information about impact of trial participation on your work? - To what extent was information about potential drug benefits important? - To what extent were you concerned about drug side effects/risks? - What other information would have been helpful? - How were they involved? - Who did you not want involved in your decision? - Who made the final decision? - How comfortable were you with your involvement in the decision? - What would you do differently in the future? |
| 1. Barriers and Facilitators | - What were some of the challenges you faced in your decision making? What supported you in your decision-making? - [For trial acceptors] Were there any decisions you had to make while on the trial that were challenging? - How do you feel the clinical trial recruitment process supported or hindered you in making a decision? - How could the clinical trial recruitment process be improved? - Overall, what is your opinion of early-phase trials? About cancer clinical trials in general? About cancer patients taking part in clinical trials? | - Resources? Information? Individuals involved? Timing? |
| 1. Final comments | - Thank you for participating in this study. Is there anything else that you would like to share about your experience in making decisions about clinical trial participant? |  |
