# Supplementary material for: Decision-making and autonomy among participants in early-phase cancer immunotherapy trials: a qualitative study
Source: BMC Cancer. 2024 Mar 25;24:373. doi: 10.1186/s12885-024-12119-7 (PMC10962144; doi:10.1186/s12885-024-12119-7)
Supplement: Supplementary file 2 — Supplementary Material 2: Participant Characteristics [file 12885_2024_12119_MOESM2_ESM.docx]

Table S1. Participant characteristics

| **Characteristic** | **N=21** |
| --- | --- |
| Age  Below 30 years old  40-49 years old  50-59 years old  60-69 years old  70-79 years old  80 years and older | 2 (9.5%)  1 (4.8%)  4 (19%)  9 (42.9%)  4 (19%)  1 (4.8%) |
| Gender  Male  Female | 12 (57.1%)  9 (42.9%) |
| Cancer type  Lung  Breast  AML  Ovarian  Melanoma  Sarcoma  Colon  Pancreatic  Leukemia  Cervical | 7 (33.3%)  1 (4.8%)  2 (9.5%)  1 (4.8%)  1 (4.8%)  1 (4.8%)  4 (19%)  2 (9.5%)  1 (4.8%)  1 (4.8%) |
| Education  High school diploma  College or trade school  University education or above | 2 (9.5%)  8 (38.1%)  11 (52.4%) |
| Marital status  Married/Life partner  Divorced/Separated  Never married | 16 (76.2%)  2 (9.5%)  3 (14.3%) |
| Ethnicity  White  East/South Asian  Prefer not to answer | 16 (76.2%)  4 (19%)  1 (4.8%) |
| Annual net income  Below $30,000  $30,000 - $59,999  $60,000 - $99,999  $100,000 - $199,999  Greater than $200,000  Prefer not to answer | 2 (9.5%)  8 (38.1%)  2 (9.5%)  3 (14.3%)  1 (4.8%)  5 (23.8%) |
