# Supplementary material for: Decision-making and autonomy among participants in early-phase cancer immunotherapy trials: a qualitative study
Source: BMC Cancer. 2024 Mar 25;24:373. doi: 10.1186/s12885-024-12119-7 (PMC10962144; doi:10.1186/s12885-024-12119-7)
Supplement: Supplementary file 3 — Supplementary Material 3: Themes and Supportive Quotes [file 12885_2024_12119_MOESM3_ESM.docx]

**BMC Cancer Tables Supplementary Material - 2**

**S2.a Participants’ perceptions of choice**

| Theme | Illustrative quotes |
| --- | --- |
| 1. Trial participation as an act of desperation 2. Trial participation as an opportunity | - “…they tell you clinical trials are very low [success] but what if I am in that one percent [where it is successful]… So I've got to take a chance… my options were basically zero or try, right?” (P001) - “So, when [Doctor X] said there was no other options I'm thinking there was no other options being, there's no other trial for me… and there is nothing else we can do." (P011) - “Part of my willingness [to go on the trial] is that I am toast. So why not? Go for it… it was made plain to me that I was not in good shape.” (P016) - “The bottom line is I didn't think that I had any choice. There might not be other options for me. All that might be left is palliative treatment.” (P018) - “Even though it's a Phase I [trial], it still gives you a chance. It gives you more to...to carry on and if it works, that's even better. Why throw in the towel…” (P011) - “It felt pretty lucky to be [on the trial] because it's an international trial and there's only like 400 or 500 participants and only five in Canada and I'm the second one to get it. So, I felt pretty lucky.” (P015) - “Believe it or not, I know you've been handed a terrible diagnosis. Is your life over? No because I am getting this treatment.” (P021) |

**S2.b Relational Influences on Early-Phase Trial Decision Making**

| Theme | Illustrative quotes |
| --- | --- |
| 1. Being provided with some hope 2. Having Trust 3. Having the ability to withdraw | - “It was the success rate [of the trial]. I'm not so in tune with the information but people having a very good success with it in terms of people living ten to even twenty years beyond.” (P002) - “They clearly explained why the hospital was involved and their beliefs in it and their hopes for it. He didn't offer any guarantees, right? Or anything like that but they saw potential hope.” (P013) - “They [clinical trial staff] explained that they're good possibilities, good indicators for as far as they could tell from other people that started earlier than I did [on that trial] and it's a go [for me].” (P014) - “So, at the time I thought clinical trials were basically the end of the road, when there was nothing else available then you kind of went for last resort and that could be clinical trials. So I was a little distressed… then we talked to a friend who is also a nurse who did a clinical trial for breast cancer and she said it is absolutely not a last resort. And if I had the chance to go on a clinical trial I would jump at it because it's the most, kind of leading edge research and clearly it offers you some hope”. (P020) - “I trust [Doctor X] and he believes that [this trial] is probably the best choice for me… [that trust] is partly based on my previous experience with my doctor. He’s never lied to me. He is straight forward and I like that. Some doctors would not be this straight forward.” (P009) - “This [drug] has already been cleared by the FDA's front line… Basically, it came down to...it was stupid for me not to try treatment… at that point, I had already developed a great deal of trust in my team.” (P013) - “I've had some very detailed discussions with the physician from [Cancer Centre X]… and now I read that [Cancer Centre X] I think they call it one of the best places in the world [for treatment]… I'm in their care and they do excellent care there. So, I mean, I couldn't even consider it [without that].” (P017) - “The bottom line is I didn't think that I had any choice. If my oncologist said to me that the chances of me living longer than six months were good, I gave them credibility, which is what I did. I didn't give it a second thought. If I didn't have confidence in the people that were treating me then I wouldn't have done it”. (P018) - “I know I can say no later. So, I went home and did some research and my son-in-law who is a physician associated with the University of X Hospital and he told me that yes, the trial is promising. So I said, [let’s] go ahead.” (P009) - “It was stupid for me not to try [the trial]. It's what I said to you earlier because I could always bail out of it if the treatment was worse than the disease.” (P013) - “I wanted to know whether I could quit at any time…They couldn't guarantee anything of course and I understood that.” (P014) - “It's nice to know that I could have walked away [from the trial] at any time.” (P018) |
